# Developing leadership competencies for genomics integration through globally networked learning and education

**DOI:** 10.3389/fmed.2024.1404741

**Published:** 2024-08-12

**Authors:** Jacqueline Limoges, Arja Halkoaho, Mari Laaksonen, Muskaan Karwal

**Affiliations:** ^1^Faculty of Health Disciplines, Athabasca University, Athabasca, AB, Canada; ^2^School of Health Care and Social Services Education and R&D, Tampere University of Applied Sciences, Tampere, Finland

**Keywords:** genomics, nurses, leadership, competencies, education

## Abstract

**Aim:**

To describe the implementation and evaluation of an education strategy called the globally networked learning-genomics (GNL-G) used in Master’s courses in Canada and Finland. The study focused on the feasibility and effectiveness of GNL-G in developing leadership competencies for integrating genomics into practice.

**Methods:**

Interpretive description qualitative methodology was used to explore how GNL-G, global exchanges and assignments standardized with the Assessment of Strategies to Integrate Genomics in Nursing (ASIGN) tool influenced competency development. The Reporting Item Standards for Education and its Evaluation in Genomics (RISE2 Genomics) framework guided the design, implementation, evaluation, and reporting of GNL-G. Data included one-to-one interviews, written assignments, and reflections.

**Results:**

Interviews and assignment data from three cohorts of students for a total of ten Canadian and 11 Finnish master’s students participated in this study. The ASIGN Tool played a crucial role in facilitating students’ analysis of their practice context and the development of leadership strategies. Participation in GNL-G enhanced students’ confidence to lead efforts to integrate genomics, irrespective of their genomics expertise. Engagement with global peers emphasized the importance of incorporating equity, ethics, and social justice into leadership strategies for genomics integration.

**Conclusion:**

The GNL-G strategy enhanced leadership competencies for genomics integration in graduate students from Canada and Finland. The ASIGN Tool and global peer collaboration highlight the importance of innovative educational methods in preparing leaders for the complexities of genomics in healthcare.

## 1 Introduction

The integration of genomics into all domains of nursing practice (direct care, education, administration, research and policy) requires coordinated and collaborative strategies by leaders who understand the complexities of genomic technologies and the unique challenges associated with integrating this knowledge (1–4). Leadership is needed to accelerate genomics-informed education (4–7), to design and implement practice changes driven by scientific advancements (4, 5, 8), and to navigate the new ethical, safety, and equity issues arising in the genomic era (9–11). To address these needs, nurses in formal and informal roles require leadership competency development (4, 5). While leadership is commonly cited as a critical enabler for integrating genomics, research addressing educational strategies to teach leadership competencies specific to the genomics era could not be located.

The integration of genomics is recognized as a global challenge that requires leadership support (1, 2). Given the global nature of this challenge and efforts by groups such as the Global Genomics Nurses Alliance to support leadership, we created an educational strategy that linked nurses from two countries to enhance global networking and collaboration, and to use the strengths of international nurses in genomics. In this article, we report on a study exploring the impact of an educational strategy called globally networked learning-genomics (GNL-G) that was implemented with Master’s students in Canada and Finland to prepare the next generation of genomic leaders. This project was formed out of an international collaboration between JL and AH who share mutual goals for developing educational activities to promote leadership competencies. Furthermore, Canadian and Finnish nurses are in the infancy stage of workforce development for the genomic era (6, 12), both countries have publicly funded and accessible health systems, and similar nursing education credentialing requirements. As such, students from Canada and Finland were considered good partners for exploring this educational intervention.

The gaps in research examining how educational strategies address learning objectives are a known barrier to advancing the genomics nursing workforce (13). Standardized frameworks are recommended to develop evidence for best practices in education, and to accelerate the integration of knowledge into practice (14). Standardization in design and evaluation can provide structures that aid the appraisal and replicability of the intervention and enhance global initiatives (1). Therefore, to promote pedagogical advancement, the teaching and learning strategy (GNL-G) and its evaluation were designed using the criteria suggested in the *Reporting Item Standards for Education and its Evaluation in Genomics* (RISE2 Genomics) framework (14). There are 31 standards in the RISE2 Genomics Framework, and an important standard is the design of the strategy to align with the learning objectives and another is the systematic evaluation of the learning strategy against the learning objectives (14).

### 1.1 Aim and research questions

The aim of this study was to implement and evaluate an educational strategy called globally networked learning-genomics (GNL-G) in graduate education courses in Canada and Finland. The following research questions guided this study: (1) Is GNL-G a feasible and helpful teaching and learning strategy to develop leadership competencies associated with integrating genomics into practice? (2) What are the experiences of graduate students who use GNL-G and the ASIGN Tool for developing leadership competencies associated with integrating genomics into practice?

## 2 Materials and methods

### 2.1 Design

To design the educational strategy, consideration was given to the global networking and supports that currently exist for the integration of genomics and how this could be used in graduate education. Distance education using computer conferencing to support learning about leadership, healthcare, and global issues influencing nursing has a long history of over 20 years (15). Globally networked learning (GNL) is a term commonly used to describe an approach to education that uses internet and computer technology rather than geographic mobility for global exchanges in learning (16). GNL has been used to support education on concepts related to leadership such as learning about diverse people and nursing practice (16, 17). It has been used to assist students in understanding international aspects of nursing, to build global professional relationships, and to explore how nursing care can be modified to meet the healthcare needs of diverse populations (18–21). Online learning has also helped students to connect internationally, appreciate cultural differences and commonalities, recognize assumptions, and explore solutions to common professional challenges (22, 23). Given this evidence, we decided to use GNL to structure our approach to delivering education on leadership and the integration of genomics. Using GNL allowed us to leverage the growing strength in genomics literacy and genomics-informed practice among nurses in different countries and to encourage global collaboration.

To develop the learning activities and assignments, and to assist with focus, standardization and replicability of the GNL-G strategy, the *Assessment of Strategies to Integrate Genomics in Nursing* (ASIGN) tool (24) was used. The ASIGN tool includes six critical success factors known to support the integration of genomics across nursing. The six factors represent essential elements that need to be in place for nurses to deliver safe genomics-informed care. The six factors are enhanced education and workforce development, effective nursing practice, infrastructure and resources that support the incorporation of genomics in practice, interprofessional collaboration and communication; family- and community-focused care, and healthcare transformed through policy and leadership. Key enablers and indicators for each of the six critical success factors are included in the tool. The tool prompts the user to determine where the practice setting is positioned on a maturity matrix relative to each critical success factor against a 5-point ordinal scale (pre-contemplation to leading). The possibility of using the ASIGN tool and the maturity matrix to guide the assessment and strategic integration of genomics within and between countries was a goal of the international research team who developed the tool (24). MEDLINE and CINAHL databases were searched, and no prior research on the use of GNL or the ASIGN tool for the development of leadership strategies targeting the integration of genomics was located, adding to the innovativeness of this strategy.

Interpretive Description qualitative methodology (25) was used to answer the research questions and to explore the impact of GNL-G on leadership development. Interpretive Description methods (25) are appropriate for applied research and are particularly well suited to research questions focused on practical knowledge development and application in nursing (25). Interpretive Description supports the analysis of qualitative data related to complex experiences such as learning to find recurrent and shared patterns that help identify disciplinary knowledge (26). This pragmatic research approach assists in developing knowledge for a practice discipline such as nursing and was considered suitable given the goals and objectives of this research project. Further, Interpretive Description is appropriate for this study given the impact of genomics in society, healthcare, nursing education and practice and how these may intersect with personal views.

### 2.2 Guiding frameworks

Our study was informed by our “theoretical scaffolding” (25), which includes our disciplinary epistemological positioning as a team of nurse researchers and educators leading the integration of genomics in our respective countries, and the existing global literature surrounding the education of the nursing workforce for the integration of genomics in nursing practice. The RISE2 Genomics framework (14) guided the design, implementation and evaluation of GNL-G and the RISE2 Genomic framework and the Standards for reporting qualitative research (SRQR) were used to guide the writing of this paper (27).

### 2.3 The GNL-G education intervention

GNL-G was used with three cohorts of graduate students in Finland and Canada over three semesters (Winter 2022, Fall 2022, and Winter 2023). Students formed dyads, matched as much as possible by area of practice such as community or hospital-based, with one student from Canada and one from Finland. The project began with an orientation about GNL-G, the ASIGN tool, and assignment requirements. Students independently used the ASIGN tool to assess their practice setting along the continuum of the maturity matrix and to identify where they would focus their leadership initiative aimed at accelerating genomics-informed practice. Within their dyads, students compared their assessment findings from using the ASIGN tool and shared their chosen leadership strategy. Each dyad presented their strategies and experiences in an exchange symposium at the end of each term. Students independently completed a personal reflection and an assignment describing their assessment, leadership strategy, implementation, and evaluation plans. The same assignment was given to the three cohorts to assess replicability. The learning activities and learning objectives which help describe the GNL-G approach are presented in Table 1.

**TABLE 1:** Description of the assignment given to learners and the learning objectives.

Learning activity	Learning objective
1. Introductions and planning (in dyad):	Learning objective #1—To get acquainted and decide how they will each use the ASIGN tool.
2. Assessing the practice setting and local context with the ASIGN tool (individual):	Learning objective #2—To identify areas (in the practice setting) for development that would support the integration and use of genomics in nursing practice.
3. Comparing and contrasting geographic differences (in dyad):	Learning objective #3—To develop global awareness of the healthcare system and nursing in Canada and Finland to explore how context influences the adoption of innovation and the choice of leadership strategy.
4. Developing a strategy to support change and practice transformation (individual):	Learning objective #4—To create one evidence-based leadership strategy that will address one critical success factor and support genomics-informed practice.
5. Sharing strategies (group): Participate in an exchange symposium with all faculty and students involved in the project to share your strategy and compare reactions and outcomes.	
6. Assignment: Create an individual slide presentation of the leadership strategy and a 500-word reflection on the learning experiences and how they influenced leadership development in support of genomics-informed care as evidence of learning.	

### 2.4 Ethical considerations

The researchers in Canada obtained approval to collect data from an ethics review board (file 24714). The researchers from Finland received approval from their university. In both countries, student participants provided informed consent to be interviewed and to have their assignments and reflections analyzed as research data. Also, written informed consent was obtained from the students for the publication of any potentially identifiable images or data included in this article.

Students were assured that their decision to participate would not affect their studies or evaluations.

### 2.5 Study setting and recruitment

At the end of each of the three semesters (Winter 2022, Fall 2022, and Winter 2023) and after all course grades were posted, participants for this study were recruited from all of the students who completed the GNL-G project. Students were sent an email inviting them to join the study. Our goal had been to form three dyads in each of three semesters, but in two of the semesters, there was greater interest, and thus, we formed four dyads in those semesters. In total, 22 students completed GNL-G and 21 students agreed to participate in this research.

### 2.6 Inclusion and exclusion criteria

Participants were included in this study if they were Master’s students who had participated in the GNL-G strategy that was implemented in either JL or AH’s course. This was a convenience sample with no other inclusion or exclusion criteria.

### 2.7 Data collection

Individual semi-structured interviews using an interview guide (see Table 2) were conducted by the faculty researchers (JL and AH) to obtain first-hand accounts of experiences and the impact of GNL-G on meeting the learning objectives. At the start of the interview, students were encouraged to provide their perspectives and to speak freely about their experiences. These strategies were used to limit the power imbalance between the faculty and students. The one-to-one interviews were recorded and transcribed. All participants agreed to the use of their assignments as study data and these assignments were downloaded from the online course and anonymized. Given the language barriers, the researchers collected and analyzed data for the students in their country (e.g., JL and MK for Canada and AH and ML for Finland).

**TABLE 2:** Semi-structured interview questions.

1. Please tell me about your experiences with the globally networked learning project.
2. What did you learn from this experience?
3. How did interacting with students from another country influence your understanding of leadership, genomics, and strategies to support the integration of genomics into practice?
4. How did you use the ASIGN tool? What needs did you identify, and how did you decide what to focus on?
5. How do you think GNL and this assignment contributed to (a) your leadership skills, (b) your understanding of genomics, and (c) how culture influences leadership and diffusion of innovation?
6. What are your views on the seminar?
7. What recommendations do you have for improving the assignments (so that they further support your learning)?
8. What strategies did you use to connect/communicate with your partner? How did that impact your learning?

### 2.8 Rigor and reflexivity

We maintained rigor by adhering to Thorne’s (25) evaluative criteria in Interpretive Description, including epistemological integrity, by ensuring alignment between the research questions, data collection, analysis, and study goals. The research team addressed rigor by conducting an independent review of the data to determine findings and then comparing these with the other researchers on the team. Then, comparisons were made between teams. We verified our findings through research team debriefing, detailed engagement with the data, and use of participant quotes and through a robust audit trail. Author AH translated the Finnish students’ quotes in the findings section into English.

### 2.9 Data analysis

After each interview, the research team wrote reflective notes to support the analysis. The purpose of exploratory studies is not to completely describe all aspects of a phenomenon; therefore, when we assessed that there was enough information power, variation, complexity, and richness in the data to inform practice, we concluded the data collection after the three cohorts (28). The interview data were used to explore learning experiences and how GNL-G helped meet the learning objectives. The assignments were used to provide further evidence of the students’ ability to meet the learning outcomes, use the ASIGN tool, and develop targeted leadership strategies to address a known critical success factor for the integration of genomics.

The interview transcripts and assignments were read and analyzed using content and thematic analysis using Interpretive Description approaches described by Thorne (25). We first identified different organizing structures to conceptualize the data. We identified the best organizing structure by considering the goals of the study, our intended audience, and the RISE2 Genomics framework (14). Given the language differences, JL and MK analyzed the Canadian which was in English, and AH and ML analyzed the Finnish data. Each researcher read, analyzed, and coded data independently to identify common experiences and the degree to which students achieved the course learning objectives. Afterward, JL and MK analyzed the Canadian data together, and AH and ML analyzed the Finnish data to discuss themes, social processes shaping experience and to examine the quotes that would be used to support the themes. Finally, the entire research team met to pull the analysis together to discuss themes and social processes shaping experiences. Commonalities and places of divergence were discussed until consensus on the findings was achieved. The sample was small, so we did not use qualitative research coding software.

## 3 Results

Ten Canadian and 11 Finnish master’s students participated in this study. The 10 Canadian students were registered nurses seeking a master’s degree in either health sciences or nursing. Ten of the Finnish students were registered nurses, and one was a medical laboratory scientist. All Finnish students were seeking a master’s degree in either Health Promotion or Genetic and Genomic Counseling. Students worked as healthcare providers in a variety of healthcare settings.

Overwhelmingly, the students indicated that they benefitted from engaging in the GNL-G project and felt it added an interesting dimension to the course that enhanced their learning and level of engagement. The findings are organized into two parts. The first details the use of the structured assignment with the ASIGN tool and how this impacted the learner experience, ability to meet learning objectives, and replicability of the assignment between two countries. The second section describes the experiences with GNL-G and how this helped students meet the identified learning objectives, including developing leadership competencies for the genomic era.

### 3.1 The impact of the ASIGN tool in the assignment

Students identified the ASIGN tool as a key enabler to their learning. They explained how they successfully used it to examine their practice context for critical success factors and identify a target for their leadership strategy. Participant #5 (Finland) stated: “*The matrix [ASIGN] was good as it showed where I could put emphasis on something concrete.*” They used the ASIGN tool to understand where their practice area was positioned along the continuum of the maturity matrix, such as the pre-contemplation or active commitment stage, which provided a vision for achievable goals and ideas for a leadership strategy. As explained by Participant #4 (Canada):

*We could see what we were striving toward and what we didn’t have, so we knew where to start… If we were at an awareness stage or implementation stage of the maturity matrix, we would have to create pathways on the units that would allow genomics to be brought into the practice setting. It was not overwhelming; it really made it feel like something that could be accomplished, which is a big thing to say for something like genomics that I don’t know anything about.*

Students explained how the ASIGN tool assisted them to understand the stages to implementation, and to consider antecedents and factors driving success. For instance, one participant explained how initially, her goal was to implement genomics into her curriculum. After using the ASIGN tool, she could see other aspects or precursors attached to this goal. As such, in her assignment, she identified leadership strategies that would build awareness and assist her team in moving toward contemplating the importance of genomics as the faculty did not yet recognize the relevance of genomics to nursing practice or patient health objectives. She could see how smaller steps were needed before implementing genomics in the curriculum. Another Participant 8 (Canada) stated:

*The ASIGN tool was great. It was the first time I’d worked with the maturity matrix, and it really broke the factors into sections of different areas and perspectives to consider from a leadership angle. So, the tool helped identify practical support needed and where we actually are with awareness [of genomics].*

The positive influence of the ASIGN tool was detected in the assignments, where each student described the results of the assessment of their practice setting based on the ASIGN tool, identified a critical success factor that could be enhanced by leadership strategies, and indicators that would suggest improvement along the maturity matrix. Each learner created a targeted leadership strategy suitable to their practice setting and aligned to the position on the maturity matrix to address a known critical success factor. Participant 1 (Canada) relayed her experience: “*I felt like we were actually developing material that was important and got people thinking in a way that would make progress, which was kind of exciting.*” Using the ASIGN tool, most students noted that their practice setting was at the pre-contemplation phase of implementation. They identified the critical success factor to genomics integration as enhanced education and workforce development and a key enabler as a positive attitude toward genomics. Many mentioned the key indicator of nurse leaders and managers recognizing the roles of nurses in genomics healthcare. As such, many chose awareness-raising activities to help move the workforce in their practice area from the pre-contemplation to the awareness and planning stage of maturity through education. In their reflection assignments, students wrote how they felt prepared for leadership roles in the genomic era as a result of participation in the GNL-G.

Although the students perceived the ASIGN tool as challenging at first, due to the number of critical success factors and their unfamiliarity with maturity matrix tools, they persisted in learning how to use it and found it useful. The ASIGN tool also helped them to collaborate and support each other, as they had a common focus on their work. Participant 5 (Finland) stated:

*It wasn’t very straightforward; I read the article several times, gradually grasping the content. My Canadian partner agreed; the measure was probably good, and the matrix was useful for putting concrete things into it. I think it initially produced a main idea, easy to talk about in everyday language. However, when it had to be placed in the matrix, it wasn’t as simple anymore, as there were so many points. But gradually, it became easier.*

An unexpected advantage of using the ASIGN tool was how it assisted students to recognize that they did not need to be experts in genomics to lead in the genomic era. None of the students had a background in genomics. Initially, they found themselves immersed in trying to become subject matter experts in genomics until they realized this was not necessary—that foundational knowledge in genomics was adequate to lead implementation strategies. This was a significant turning point in their leadership development as they felt that otherwise, their perceived low levels of genomic literacy would have been a barrier to engaging in any leadership initiative related to this topic. The ASIGN tool helped the students to focus on developing leadership strategies to address a critical success factor for the integration of genomics and to understand more about implementation of new technologies and knowledge. Participant #4 (Finland) stated: “*It was really helpful because it is not just a vague tool, it actually has action items that you can do.*” Overall, the ASIGN tool assisted students in understanding their practice context, the readiness to integrate genomics and to identify a suitable strategy that aligned with the state of readiness of their practice context. The tool allowed students to concretely identify the stage of genomic integration, making it possible for these novice leaders to consider which specific areas to prioritize to advance the implementation of genomics into practice.

Students consistently said the ASIGN tool standardized the assignment which enabled them to collaborate across geographic boundaries. They attributed the ASIGN tool to helping them develop a global awareness of health systems and explore how context influences the adoption of innovation. Furthermore, students indicated that the assignment expectations which were based on using the ASIGN tool were clearly laid out and aligned with the learning objectives.

### 3.2 GNL-G assignments and networking

Students indicated that the effort to create the learning activity, partnering with learners from another country, and engaging in the research on GNL-G highlighted the importance and urgency of addressing leadership for the genomic era. All students stated that the extra attention by faculty, the topic of genomics, and the sense that they were representing their country added interest and enhanced engagement. Participant #2 (Canada) provided this insight: “*…right from the initial readings that you sent us, I caught that sense of urgency that this is something that’s really important and how finding an actual solution was important because it wasn’t a hypothetical problem.*” There was an appreciation for the fact that GNL-G was being evaluated and that they would be part of a project generating evidence for effective teaching and learning related to genomics.

Students explained how the coordinated learning activities and assignment requirements specifically assisted them to improve their leadership skills and consider the application of genomics across the healthcare continuum. A participant from Finland (8) described their key learnings: “*Despite not holding a managerial position, I acquired valuable insights into functioning as a project or team leader. My knowledge of the challenges associated with leadership has increased, fostering a deepened understanding and awareness of areas for improvement [to support genomics integration].*”

Participation in GNL-G bolstered the students’ confidence to take on leadership roles in the integration of genomics-informed care, even if they were not content experts in genomics or experienced leaders. Students realized they could learn about genomics and liaise with subject matter experts as needed, thus enabling them to focus on leadership strategies. They learned enough about genomics to understand how it was specifically relevant to their practice area, how it is a complex knowledge form with unique social and ethical concerns, and the importance of concerted leadership strategies to support implementation. Participant #8 (Canada) stated: “*I did find it difficult to put the two ideas [leadership and genomics] together at first. But when I reflected on it, I could see how they fit together to support changes in my approach*”. As a Finnish student (participant #9) highlighted, “*I’m in the early stages of my studies, and genome care and leadership are new things to me, … yet my knowledge of the genome deepened.*”

Liaising with students in another country allowed the participants to discern the magnitude of the gaps impeding the integration of genomics and how this is a global nursing challenge. The quote by Participant #9 (Canada) illustrates this point: “*It caught me off guard to see that there could be something this massive that I didn’t know anything about.*” They also expanded their understanding of the importance of leadership and the strength of global collaboration. Students relayed comfort in knowing that nurses in other countries also struggled with the integration of genomics. Participant #2 (Finland) echoed this idea:

*It was interesting to learn more about Canada’s situation and realize how experiences and situations are similar. Globally networked learning allowed us to learn together and direct interventions in the same direction while considering regional differences. GNL offers advantages as we can learn from each other about what could be done differently to foster progress. This type of collaboration was a new experience for me, and I am delighted to have been a part of it.*

The Canadian students thought the Finnish students would be more advanced in the integration of genomics and vice-versa. However, they learned that they were all in the early stages of adoption and that concerted strategies were needed universally. Participant #8 (Canada) shared this view: “*I understand how little [genomics] is in primary healthcare and how little it is taken into account in patient care. It should be used more. I noticed this was the same with my partner.*” The intercultural exchange enhanced learning on how the practice context impacts the adoption of genomics and increased their awareness of genomics across diverse professional settings.

Students indicated that GNL-G assisted them in understanding the complexity of genomics as a knowledge form and in understanding the steps needed for the safe integration of genomics into practice. The opportunity to explore equity, ethics, and social justice with a student in another country was impactful. Students learned about the importance of designing leadership strategies that include specific consideration of equity to ensure that these topics are addressed during the integration of genomics into healthcare. Comprehending disparities that transcended the geography of Canada and Finland elicited interest and motivation to address the root causes of inequity. The intercultural learning promoted an understanding of historical and current practices that impact genomics, such as the treatment of Indigenous peoples and experiments conducted during World War II. The opportunity to talk about these various historical and ongoing processes that impact attitudes and uptake of genomics by different communities impacted learners. Participant #3 Canada stated:

*When we got into talking about social justice and equity… I brought up the idea of making sure that any barriers to Indigenous people accessing genomics, such as language, geographical, or socioeconomic barriers, are considered. So, even though we were so far apart, we were able to make connections like that.*

Similarly, a Participant (#11) from Finland explained: “*Culture has an impact [on the integration of genomics] in many ways. When discussing this with my partner, various aspects were compared, such as adequate living arrangements, heritage, and culture, and how these influence how people can and do handle things.*” Students relayed their growing understanding of the equity and ethical issues associated with genomics and their view that this would not have been possible without the global exchange.

Participants recognized that GNL-G facilitated an expansion of their leadership competencies and the importance of concerted leadership strategies for integrating genomics into healthcare. Participants attributed the activities within the GNL-G to their high level of engagement in learning. Participant #1 (Canada) said: “*I found it extremely rewarding, and I enjoyed listening to the presentations at the end.*” Another participant #4 (Canada): “*It’s not every day that I learned that much or that I get that excited for an assignment, so it was good!*” Even though students found genomics to be a new and challenging concept, integrating it into leadership was seen as highly positive, expanding the vision of the future of healthcare. Participant #1 (Finland) stated: “*All of our leaders and supervisors should receive training on genomics awareness and its advancement. If we can convince nursing leaders about this, then we could have them share this awareness with their teams to promote a positive culture that includes genomics.*”

## 4 Discussion

The design of the GNL-G intervention, evaluation, and reporting were structured using the 31 items from the RISE2 Genomics framework (14). As such, we have evidence of how GNL-G was standardized, replicated and contributed to meeting the learning objectives (see Table 1) and developing leadership competencies for the integration of genomics into practice in two countries. The students in this study reported that GNL-G positively impacted their leadership development and identified that using the ASIGN tool (24) was a key contributor to this experience. Developing leadership competencies to narrow the gap between scientific advancements and genomics-informed practice is essential to successful implementation strategies (29–31). Students explained how the ASIGN tool and the global networking enabled them to assess their practice setting and design leadership strategies aligned with known critical success factors to accelerate the integration of genomics. Employing the ASIGN tool assisted students to realize they did not need to be genomics experts to identify strategies to support integration or to lead. By focusing on the critical success factors and the practice context, learners felt empowered to engage in leadership strategies.

The global exchange of ideas enhanced learner engagement as students felt they were representing their country and felt responsible for sharing appropriate knowledge about social and health disparities and how the health system was structured to address these. Similar reports were made by undergraduate students involved in GNL (16) which emphasizes these types of benefits from global learning. For example, the global exchange assisted students to recognize genomics as a complex knowledge form with important scientific, equity and social concerns. Students recognized that while people in their countries had experienced different historical events such as WWII and colonization, these events continue to impact the uptake of genomic technologies. These conversations amplified their awareness of health, equity, and diversity and reinforced their view that these topics must be considered when leading genomics implementation strategies. Given the growing understanding of health disparities arising from the integration of genomics (32), ensuring leadership development incorporates these critical elements is vital to ensuring that genomics benefits all. Increased understanding of international aspects of nursing, how to build global professional relationships and how nursing care can be modified to meet the healthcare needs of diverse populations have been reported with global web-based learning (18–20, 22). The mounting evidence highlights additional benefits from globally networked learning strategies.

Nurses’ engagement in genomics has remained a global challenge (1, 2, 33, 34) and support is urgently needed to address the longstanding barriers to genomics-informed nursing practice (2). Challenges such as a lack of support or opportunities to integrate genomics into education and practice (35, 36), and the lack of an effective policy infrastructure (37) are all amenable to leadership strategies. Assisting emerging leaders to recognize the role of workforce development, implementation science, change management, and inter-professional collaboration requirements is crucial to the safe and equitable integration of genomics (4–6). This study shows that students can assess critical success factors using the ASIGN tool (24) and generate leadership strategies to support the integration of genomics into practice. A prepared leader can support the development of genomic literacy to promote equitable and accessible genomics-informed healthcare (10, 38) and augment nurses’ contributions to genomic healthcare (9).

### 4.1 Limitations of the study

A limitation of this study is that all research team members could not collect or read all study data due to language barriers. This could limit the rigor of the analysis. We anticipated this situation as JL and AH had previously conducted international research projects. Thus, we formed a research team with two researchers in each country who could analyze the data to support rigor. We also used discussions with the entire team when identifying themes and quotes and returned to the data to ensure accuracy. While there is significant value in international research, language barriers can present unique challenges that can generate limitations. With Interpretive Description, there is no set sample size, however, we recognize the diversity between Canadian and Finnish students and healthcare contexts and that our sample of 21 students could limit the understanding of the many complexities we explored.

### 4.2 Recommendations for further research

To enhance the continued exploration and adoption of GNL-G, further research should focus on several key areas. Longitudinal studies can track the sustained impact of the leadership competencies developed through GNL-G and aid in understanding how these competencies support progression on the maturity matrix in real-world settings over time. Additionally, integrating GNL-G into various educational settings and in different countries will assist in assessing its effectiveness in enhancing leadership skills among diverse students. Addressing these research areas will provide robust evidence of the usefulness of GNL-G in developing leadership competencies and could open new avenues and areas of research.

### 4.3 Implications for practice

Teaching leadership strategies, guided by tools such as the ASIGN, can assist emerging leaders to address known critical success factors in the integration of genomics and prepare them for leadership roles. The study underscores the importance of prepared leadership for equitable genomics-informed healthcare. Employing the RISE2 Genomics framework in the design, implementation, and evaluation of GNL-G assisted with standardization, replicability across countries and cohorts, and in understanding how the GNL-G contributed to learning outcomes.

## 5 Conclusion

Using the RISE2 Genomics framework at the outset of this project ensured that the 31 items to standardize education design, implementation, and evaluation were considered and addressed. GNL-G can be replicated and researched to build evidence for nursing education. Evaluating the GNL-G against the learning objectives enabled an understanding of how GNL-G, which included using the ASIGN tool, global networking, and the assignment, contributed to meeting the learning objectives. The ASIGN tool was effectively used to assess the practice context for critical success factors and to create a targeted leadership strategy to enhance genomics-informed care. Participation in the GNL-G project enhanced learners’ confidence and abilities to provide leadership in support of genomics-informed care, which has the potential to accelerate the mainstreaming of genomics. Given the global challenges with the safe and equitable integration of genomics, sharing resources between countries, including lower- and middle-income countries using web-based learning strategies should be further explored to optimize the global nursing workforce.

## Data availability statement

The raw data supporting the conclusions of this article will be made available by the authors, without undue reservation.

## Ethics statement

The studies involving humans were approved by the Athabasca University Research Ethics Board, Tampere University of Applied Sciences administrative approval. The studies were conducted in accordance with the local legislation and institutional requirements. The participants provided their written informed consent to participate in this study. Written informed consent was obtained from the students for the publication of any potentially identifiable images or data included in this article.

## Author contributions

JL: Conceptualization, Data curation, Formal analysis, Funding acquisition, Investigation, Methodology, Project administration, Resources, Software, Supervision, Validation, Visualization, Writing – original draft, Writing – review & editing. AH: Conceptualization, Data curation, Formal analysis, Investigation, Methodology, Project administration, Resources, Software, Supervision, Validation, Visualization, Writing – original draft, Writing – review & editing. ML: Conceptualization, Data curation, Formal analysis, Writing – original draft, Writing – review & editing. MK: Data curation, Formal analysis, Writing – original draft, Writing – review & editing.
